# Epidermal Stem Cells Cultured on Collagen-Modified Chitin Membrane Induce *In Situ* Tissue Regeneration of Full-Thickness Skin Defects in Mice

**DOI:** 10.1371/journal.pone.0087557

**Published:** 2014-02-07

**Authors:** Yan Shen, Libing Dai, Xiaojian Li, Rong Liang, Guangxiong Guan, Zhi Zhang, Wenjuan Cao, Zhihe Liu, Shirley Mei, Weiguo Liang, Shennan Qin, Jiake Xu, Honghui Chen

**Affiliations:** 1 Guangzhou Institute of Traumatic Surgery, Guangzhou Red Cross Hospital Medical College, Jinan University, Guangzhou, People’s Republic of China; 2 Department of Medical Laboratory, Second Affiliated Hospital of Guangzhou Medical College, Guangzhou, People’s Republic of China; 3 School of Pathology and Laboratory Medicine, The University of Western Australia, Nedlands, Western Australia, Australia; 4 Student of Sophie Davis School of Biomedical Education, Mack Lipkin Fellowship, New York, New York, United States of America; RMIT University, Australia

## Abstract

A Large scale of full-thickness skin defects is lack of auto-grafts and which requires the engineered skin substitutes for repair and regeneration. One major obstacle in skin tissue engineering is to expand epidermal stem cells (ESCs) and develop functional substitutes. The other one is the scaffold of the ESCs. Here, we applied type I collagen-modified chitin membrane to form collagen-chitin biomimetic membrane (C-CBM), which has been proved to have a great biocompatibility and degraded totally when it was subcutaneously transplanted into rat skin. ESCs were cultured, and the resulting biofilm was used to cover full-thickness skin defects in nude mice. The transplantation of ESCs- collagen- chitn biomimetic membrane (ESCs-C-CBM) has achieved *in situ* skin regeneration. In nude mice, compared to controls with collagen-chitin biomimetic membrane (C-CBM) only, the ESCs-C-CBM group had significantly more dermatoglyphs on the skin wound 10 w after surgery, and the new skin was relatively thick, red and elastic. *In vivo* experiments showed obvious hair follicle cell proliferation in the full-thickness skin defect. Stem cell markers examination showed active ESCs in repair and regeneration of skin. The results indicate that the collagen-modified chitin membrane carry with ESCs has successfully regenerated the whole skin with all the skin appendages and function.

## Introduction

Extensive dermal and full-thickness injuries are usually related to acute incisional and excisional wound, trauma and burn [1], [2], [3] The complications of these injuries may be severe, and especially, the regeneration of a full-thickness skin is not sufficient and pertains to dermal regeneration [4], [5], [6]. Autologous split skin grafts are the gold standard for the treatment of full-thickness injuries such as extensive burns [7]. However, the limited donor grafts and tedious surgeries urge the development of proper skin substitutes through tissue engineering. Current artificial skin materials include epidermal substitute, dermal substitute and full skin substitute [8], [9], [10]. However, cells in these substitutes have a relatively weak ability to proliferate and self-renew, which affects the outcome of repair, with the results of blisters, scar hyperplasia and severe contraction. For example, epidermal substitute is made by adding epidermal cells to a scaffold to form a composite skin with some biological activity. However, the seed cells of the dermal substitute are mainly fibroblasts. Although fibroblasts are easily to obtain and grow relatively quickly, their function is simple and they do not encourage the development of skin appendages (i.e., sweat glands, hair and hair follicles) [11], [12], [13], [14]. This problem might be resolved effectively if the dermal substitute is used to cover the wound with a population of epidermal stem cells (ESC).

Stem cells demonstrate the two defining features, namely self-renewal and multipotency, and are instrumental for renewal, regeneration and repair [15]. In skin tissue engineering, ESCs have the advantages of definite orientation, great plasticity and the ability to induce and form skin appendages [16]. Therefore, they have become the first choice of seed cells in skin tissue engineering. Recent findings suggest that the hair follicle is a major repository of epidermal stem cells which give rise to several cell types of the hair follicle as well as upper follicular cells [17]. The use of ESCs with hair follicle bulge may potentially resolve many current problems in skin tissue engineering, including lack of skin appendages and immune rejection. Besides the seed cells, selection of a proper carrier for ESCs is the key to successful skin tissue engineering.

Chitin is a naturally occurring biopolymer having diversified applications not only in the pharmaceutics for drug delivery, but also in the biomedicine as dress material for wound healing applications and as potential biomaterial for tissue engineering [18], [19]. The structure and function of chitin are similar to those of mucopolysaccharides in the skin. Chitin contains an active hydroxyl (−OH) group adjacent to the amino (−NH2) group and is a hydrophilic cation polymer. The molecule is composed of acetylglucosamine and glucosamine units, and it is also a constituent of human hyaluromic acid. Chitin has excellent biocompatibility and can be degraded into chitin oligosaccharide by lysozyme, xylanase at a rate of absorption and utilization of 100% [20]. It is also a relatively good adhesive, which may enhance the proliferation and differentiation of cells on the material, promote re-epithelization in animal skin wounds, and accelerate wound healing [21]. Previous studies suggest that chitin enhances the cell proliferation and differentiation, promotes epithelization but significantly inhibits type I collagen and accelerates type III collagen secretion, and therefore accelerates skin wound healing [22]. More importantly, chitin has no immunogenicity without any risk of immune rejection after implantation [23]. These properties make it a very promising scaffold for skin tissue engineering. In our previous study [24]. a skin substitute with chitin membrane seeding with rat epidermal stem cells (ESCs) has been evaluated for its promising application in skin engineering. Chitin membrane used as the ESCs culture vehicle has a good biocompatibility. However, shortcomings remain. The pore size of the chitinous biomembrane material is relatively large (500–800 µm), the grid is uneven and the compactness poor. Therefore, it can only be used as a temporary wound dress, and is not suitable for cell adhesion and three-dimensional cell growth. Type I collagen is a good culture substrate for many cell types. It is possible to be integrated into chitin membrane to improve cell adhesion and proliferation, reduce pore size.

In the current study, the chitin membrane was modified by cross linked with type I collagen isolated from the rat tail. This collagen-chitin membrane had a great biocompatibility and degraded totally when it was subcutaneously transplanted into rat skin. The modified collagen-chitin biomimetic membrane (C-CBM) had a relative small pore size (2–10 µm), and the adhesion of hair follicle ESCs on the composited membrane was improved. Further, the isolated hair follicle ESCs were seeded to the C-CBM and transplanted into nude mouse full-thickness skin defects. The defects were fully repaired, and the regenerated skin was proved to contain all the skin appendages. Therefore, these data suggest that hair follicle ESCs carried in the modified collagen-chitin scaffold is sufficient to achieve morphological, structural and functional reconstruction of the full-thickness skin defects.

## Materials and Methods

### Ethics Statement

All experimental procedures were performed according to the Guide for the Care and Use of Laboratory Animals and were in compliance with the guidelines specified by the Chinese Heart Association policy on research animal use and the Public Health Service policy on the use of laboratory animals. The animal use protocol has been reviewed and approved by the Animal Ethical and Welfare Committee (AEWC) of Guangzhou Red Cross Hospital. SD rats and BALB/c nude mice were selected from the Experimental Animal Center of Guangdong Province. Licence : SCXK(GD)2008-0002, SCXK(GD)2011-0029, SYXK(GD) 2007 -0081.

### Materials and Equipment

The following major materials were used: chitin natural biomembrane (Kisumi, Hunan Yinghua Biomedical Co.,Changsa Hunan, China); defined keratinocyte serum-free medium (DK-SFM, 10785, Gibco, Carlsbad, CA); rat type I collagen (3-D Culture Matrix™, Trevigen, Inc., Gaithersburg, MD); type IV collagen (Sigma-Aldrich Biotechnology, L.P., St. Louis, MO); mouse anti-rat β1 integrin (CD29) monoclonal antibody, mouse anti-rat cytokeratin antibodies, anti-CK5, CK10, CK14, CK15 and CK19 antibodies, mouse anti-rat p63, vascular endothelial growth factor (VEGF) and Dsg antibodies (all Santa Cruz Biotechnology, Santa Cruz, CA); anti-CD34 and CD200 antibodies (Abcam, PLC, Cambridge, UK); diaminobenzidine chromogenic reagent (DAB, Boster Biological Technology, Ltd., Fremont, CA); quantitative polymerase chain reaction (PCR) enzyme SYBR Green PCR Master Mix (Toyobo Co., Ltd., Osaka, Japan); RQ1 RNase-free DNase (Promega M6101) Corp., Madison, WI); and MaxVision™ HRP-Polymer anti-Mouse/Rabbit Immunohistochemistry (IHC) Kit (Shenzhen Maixin Bio-tech Co., Shenzhen, China).

The following major equipment was used: real-time PCR instrument (RT-PCR, ABI PRISM® 7300 Sequence Detection System, Applied Biosystems, Foster City, CA); confocal laser scanning microscope (ZEISS LSM 510 META, Oberkochen, Germany); and scanning electron microscope (SEM, XL-30-based Environmental Scanning Electron Microscope, Philips, Hilversum, the Netherlands).

### C-CBM Preparation and Measurement

The procedures described below are carried out on ice. Sterile 10×PBS, sterile distilled water, sterile fresh 1 M NaOH solution and type I collagen were mixed to prepare 5 mg/ml type I collagen according to a volume ratio of 5 mg/ml type I collagen: 10×PBS: 1 M NaOH = 10∶5: 1. Sterile distilled water up to total volume and adjust the pH to 6.9 with 1 N HCL. After mixture, the final concentration of type I collagen was 0.5 mg/ml. A soft mixture was achieved using a suction pipette. Centrifugation (300 g×10 min) was carried out to remove any air bubbles. The collagen mixture was left to rest for 15 minutes and then the chitin membrane was modified with type I collagen. The chitin membrane was trimmed to 5 cm×5 cm. Double-layer cross-type placement of the chitin membrane was carried out according to the material texture on a mold of the same area. 5 ml type I collagen mixture was added into each mold. The mold was placed horizontally on ice for one hour and then placed in a 37°C incubator for another hour to promote colloid formation. Observation of C-CBM characteristics were done with a confocal microscope. Pre-cooling was carried out at −80°C for 20 hours or overnight. Then, freeze drying was done at −40°C for 25 hours. Co^60^ irradiation at 5 kGy was done four times for a total of 20 kGy to promote cross-linking and sterilization. Then, the membrane was observed with a confocal laser scanning microscope. Collagen-modified chitin membrane of 2–10 µm thick was obtained and the C-CBM membrane was observed under a SEM. Repeated cross-linking using Co^60^ and sterilization of C-CBM was carried out. The specimen was fixed with 2.5% glutaraldehyde overnight, and then rinsed with PBS three times. After gradient alcohol dehydration, lyophilization, gold spraying and vacuum processing, the membrane was observed with a SEM again.

### Preparation of Chitin Membrane Leaching Solution

After calculation of the surface area of the chitin membrane, 10 ml DMEM culture medium was added per square centimeter, the sample was held at 37°C for 24 h and the supernatant was obtained. FBS was added to produce a leaching solution of 10% FBS, which was the starting concentration. Gradient tests were carried out according to double dilution.

### Effect of Chitin Membrane Leaching Solution on ESCs Proliferation

Three ESCs were chosen, and the cell concentration was adjusted to 2×10^4^/ml after trypsin digestion. The solution was added to 96-well plates, 100 µl in each well and incubated in 37°C for 24 hour. The culture medium and non-adherent cells were discarded. Double-diluted leaching solution was added to make six groups: 1/1 (original leaching solution), 1/2, 1/4, 1/8, 1/16, 1/32, each group filling four wells. The control group only had culture medium added, no ESCs. After 96 hour incubation, Alamar Blue® assay was carried out, and the results were statistically analyzed.

### Isolation of ESCs, Seeding and Culture on C-CBM

Twenty clean degree 1–3 day old Sprague Dawley (SD) infant rats weighing 18–25 g were provided by the Experimental Animal Center of Sun Yat-sen University. Animal experiments were carried out in strict accordance with the established institutional guidelines for animal care and by the authors’ affiliated institutions. Full-thickness skin of the SD infant rats was harvested. ESCs were obtained by using collagen IV adherent method with Dispase II and trypsin. After removal of the subcutaneous tissue, the skin was trimmed to 2×1 cm, then digested with 0.5% dispase II overnight. Next, the skin was re-warmed for 15 minutes, and the epidermal layer was separated from the dermal layer. The epidermis was digested by 2.5% trypsin at 37°C for 10–15 min to prepare single-cell suspension. Digestion was terminated by adding culture medium containing 10% FBS. After filtration with a 200 mesh filter and centrifugation at 1000 rpm/min for 10 min, the supernatant was discarded, and the pellets re-suspended in Defined Keratinocyte-SFM (DK-SFM). Then, the solution was placed in a culture bottle pre-coated with rat type IV collagen and left at 37°C for 10–15 min. The keratinocytes within the supernatant were discarded. An appropriate amount of fresh SFM was added to the adhered cells (ESCs), which were cultured at 37°C in a 5% CO_2_ humidified incubator. The medium was changed every other day. After the cells became confluent, 0.25% trypsin and 0.02% EDTA were applied for digestion at 37°C for 10–15 min to collect cells, which were used for passage or experiment.

### Alamar Blue® Applied to Measure the Growth Curve of ESCs

Cells were placed in a 96-well plate. Before the end of culture, 20 µl Alamar Blue® dye was added. Colorimetric analysis was carried out at 4 and 7 h. The absorption values were measured directly with a microplate reader. The maximum absorption of oxidized Alamar Blue® was at 600 nm; the maximum absorption of deoxidized Alamar Blue® was at 570 nm, so that the value of deoxidized Alamar Blue® dye in living cells was obtained by subtracting *ABS*_ 570_ from *ABS*_ 600_. The color depth was proportional to the number of living cells.

### Measurement of Proliferation and Differentiation Markers of ESCs

Surface markers of ESCs, including CD29, CD71, CD49d and CD34, were examined by flow cytometry. ESCs were harvested, the cell concentration adjusted to 3×10^5^/ml, then the mixture reacted with a primary antibody at room temperature for 30 min. Next, it was washed with PBS twice, and then reacted with FITC or PE-conjugated secondary antibody for 30 min in the dark. The cells were re-suspended with PBS on ice, and then measured by flow cytometry.

### Measurement of CK15, CK19 and p63 Expression by IHC using Strept Avidin-Biotin Complex (SABC)

ESCs were placed in a mixed solution of 30% H_2_O_2_ and methanol (1∶10, v/v), soaked at room temperature for 10 min to inactivate endogenous enzymes, and washed three times with PBS for 5 min each. After heat-induced antigen retrieval and membrane rupture, 50 µl normal goat serum blocking solution was added together with anti-CK15, CK19 and p63 monoclonal antibodies. The mixture was then placed in a wet box at 4°C overnight. Next, it was placed in a 37°C box for 20 min for re-warming, and then washed three times with PBS for 3 min each time. Biotinylated secondary antibody was added and the reaction carried out at 37°C for 20 min, and then the mixture was washed with PBS three times for 3 min each time. SABC was added, the reaction carried out at 37°C for 20 min, then the mixture was washed with PBS three times for 3 min each time. DAB was added and the mixture incubated for 30 s to 5 min,and subsequently washed with distilled water. Hematoxylin counterstaining, dehydration, vitrification and mounting were then done.

### Seeding of ESCs on CBM and C-CBM *In vitro*

The modified chitin membrane was trimmed to a 10 mm×10 mm square, sterilized with ultraviolet irradiation, balanced by culture medium and preserved. Cultured ESCs were digested and rendered into suspension, and the cell concentration was adjusted to 2×10^6^/ml. Surface implantation was carried out with 0.5 ml cell suspension, which was dropped onto the surface of the modified chitin membrane and put aside for 1 h. DK-SFM containing 20% FBS was added and the membrane was placed in a 37°C, 5% CO_2_ incubator. Routine culture was applied for 1–2 w, with the medium changed every other day. When a biomimetic skin covering of ESCs-modified chitin membrane formed, it was used for measurement or animal experiment.

### Characterization of Cellular Behavior on Chitin Membranes

The states of cell growth on the material before and after modification were compared. ESCs were planted on non-modified chitin membrane and chitin membrane modified with type I rat tail collagen. Colony-formation was observed after 1–2 w in both groups. The relationship between the cells and the material and the cell spreading status was observed with a laser scanning confocal microscope. The membrane was treated described as above, and then observed with SEM.

### Establishment of Rat Full-thickness Skin Defect Animal Model and *in vivo* Degradation Test of Chitin Membrane Material

Twenty-four healthy SD rats were selected from the Laboratory Animal Center of Guangdong Province and used for both the material group and the sham-surgery (control) group. Each rat received two back wounds: one on the upper left side (the material group) and one on the lower right side (the sham-surgery group). No material was implanted in the sham-surgery group. Rats were anesthetized with 50 mg/kg 0.6% sodium pentobarbital and small incisions (about 2 cm long) were made at bilateral thick-fleshed sites of the spine about 3 cm apart. Incisions of about 1 cm×1 cm were made 1.5 cm from the spine. The incisions were deep into the subcutaneous layer to form a mechanically injured animal model. For the material group, 1 cm^2^ of chitin membrane blank material was implanted in each wound. Compressive dressing was applied. Each rat was put in a separate cage. Four specimens were harvested at 1, 2, 4, 6, 8 and 10 w each, embedded with paraffin and stained with hematoxylin and eosin (H&E) stain.

### *In situ* Induction of Epidermal Regeneration by ESCs-modified Chitin Membrane in Nude Mice Full-thickness Skin Defect Model

Sixty-three BALB/c nude mice were randomly divided three groups which were the type I collagen, experimental group (ESCs-C-CBM) and the control group (C-CBM without cells).each group divide into seven timeslot. Nude mice were anesthetized with 50 mg/kg 0.6% sodium pentobarbital and incisions made 1.5 cm from the spine to produce a full-thickness skin defect model as described above, with the upper left injury belonging to the experimental group, and the lower right injury to the control group. The type I collagen group was independent as biomaterial control. For each injury, 1 cm^2^ ESCs-C-CBM was implanted into the experimental injury and 1 cm^2^ C-CBM implanted into the control injury including the type I collagen group and C-CBM without cells group. All injuries were sutured at the four angles and compressive dressing applied. Each mouse was put in a separate cage. Three specimens were harvested at d 1 and 3, and 1, 2, 4, 6 and 10 w. H&E staining was performed and histological changes in the wound observed.

### Histological and Immunohistochemical Examinations of Paraffin-Embedded Sections

H&E staining: Paraffin-embedded sections were placed in a 55°C oven for 20 min, and xylene dewaxing carried out for 5–10 min. Then, each section was placed into a mixture of xylene and pure alcohol (1∶1) for about 5 min. Gradient alcohol hydration and hematoxylin counterstaining were performed. Color separation was carried out with 0.5–1% hydrochloric-alcohol solution. Eosin staining was performed for 1–5 min. Gradient alcohol dehydration, xylene vitrification and mounting were carried out.

### IHC Measurement of *in vivo* ESCs Markers CD34 and CD200

Each specimen was fixed with 10% neutral formalin and embedded with paraffin. A conventional tissue section was prepared and the specimen was dewaxed with xylene. Gradient alcohol hydration was performed and the specimen was placed in distilled water for later application. High-temperature and high-pressure retrieval was carried out using pH 6.0 citrate buffer at 120°C for 2 min. The specimen was washed with PBS three times for 3 min each time, incubated together with peroxidase blocking agent at room temperature for 10 min, and then washed with PBS three times for 3 min each time. Fifty µl primary anti-rat antibody CD34 or CD200 (1∶100) was added and the specimen placed at 4°C overnight, then washed with PBS three times for 5 min each time. Fifty µl HRP-Polymer anti-rat antibody was added and the specimen was incubated at room temperature for 20 min, then washed with PBS three times for 3 min each time. Coloration was carried out by adding 2 drops or 100 µl newly-prepared DAB reagent for 3–5 min. After the specimen was profusely washed with water, hematoxylin counterstaining, dehydration, vitrification and mounting were carried out.

### Western Blot Analysis of Early-stage Protein Expression in ESCs and Protein Expression of Transient Amplifying Cells (TAC) during ESCs-C-CBM Induced Skin Repair

The tissue and protein were obtained using the Trizol method. Protein sample loading buffer was added by ratio. Boiling was done for 5 min to obtain sample loading protein solution. SDS-PAGE electrophoresis was conducted as follows. Electrophoresis was done at 80 V for 40–50 min, and then electrophoresis was applied at 120 V. Low temperature membrane transfer was performed at 200 mA for 1 h. The specimen was then washed with TBST three times for 5 min each time. Five percent skim milk powder was added and the specimen was held closed at room temperature for 1 h. It was washed with TBST three times for 5 min each time, then primary antibody (1∶1000) added and the sample held at 4°C overnight. It was again washed with TBST three times for 5 min each time. Mouse-HRP secondary antibody working solution was added and the specimen was incubated at 37°C for 1 h. It was again washed with TBST three times for 5 min each time. Chamber exposure was carried out and the results were scanned and preserved. Early markers of ESCs like CD29, p63 and VEGF-A, as well as transient amplifying cell (TAC) markers CK5, CK10, CK14 and CK15 were measured by this method.

### Quantitative RT-PCR Analysis and miR-203

Quantitative RT-PCR was used to analyze the mRNA expression levels of CD29, CK15, p63 and VEGF-A throughout the ESCs-modified chitin membrane skin repair process and to track changes in the regulatory factor microRNA-203 (miR-203).

To evaluate the genetic changes of ESCs-modified chitin membrane at the early stage, the nude mice full-thickness skin defect model was examined through day 42. Primers of the target fragments were designed and synthesized according to Table 1. Specimens were harvested regularly after surgery and total RNA extraction carried out. RNase-free DNase was applied and the reaction solution was prepared, digested at 37°C for 30 min, then inactivated at 65°C for 10 min. The purity and integrity of total RNA were checked. Quantitative PCR was performed after reverse transcription. The internal control segment used was 18S rRNA-112 bp and the primer of the target fragment designed and synthesized according to Table 1. The reaction system used was 5.0 µl cDNA (1∶25) in 10 µM solution, the forward primer was 0.5 µl in 10 µM solution and the reverse primer was 0.5 µl of 2x SYBR with 10 µl Green PCR Master Mix and 4.0 µl dH_2_O, for a total volume of 20 µl. Reaction conditions were obtained by pre-denaturation at 95°C for 10 min, denaturation at 95°C for 15 s, annealing at 60°C for 15 s and extension at 72°C for 30 s. Plates were read after 40 cycles. To analyze the melting curve, the temperature ranged 60–95°C and was read every minute.

**Table 1:** Primers used in quantitative real-time PCR.

Target Fragment	Primer Design
CD29–357 bp	F:5′ACAAGAGTGCCGTGACAA
	R:5′ATTGGCACTAGCAGTGTC
CK15–271 bp	F:5′ATTCAGCAGTCAACTGGCT
	R:5′GAGCTGAGACTGCAACTCA
CK19–171 bp	F:5′ CAGCTCAGCATGAAAGC
	R:5′ GATGTCCATGAGCTGCTT
p63–383 bp	F:5′AAGCTGAGCATGTCACCGA
	R:5′TCTGATGCTGTCTTCATCTG
VEGFA-240 bp	F:5′ATGCCGGTTCCAACCAGAA
	R:5′GTGGAGGAGCGAGCTGAA

MiR-203 changes were measured simultaneously by the method described as above. The target segment was designed according to the details in Table 2.

**Table 2:** microRNA-203 Primer Design.

Target Fragmen	Primer Design
hsa-microRNA-203	F:5′ CACTCCAGCTGGGGTGAAATGTTTAGGACCA
	R:5′ CTCAACTGGTGTCGTGGA
Internal reference U6	F:5′ CTCGCTTCGGCAGCACA
	R:5′ AACGCTTCACGAATTTGCGT

### Statistics Analysis

One way ANOVA was performed using SPSS 13.0 (SPSS Inc., Chicago, IL) to analyze experimental data. The values were expressed as mean ± standard deviation (SD). A significance level of 0.05 was adopted.

## Results

### Biological Characteristics and Verification of Rat Epidermal Stem Cells

**Figure 1:**
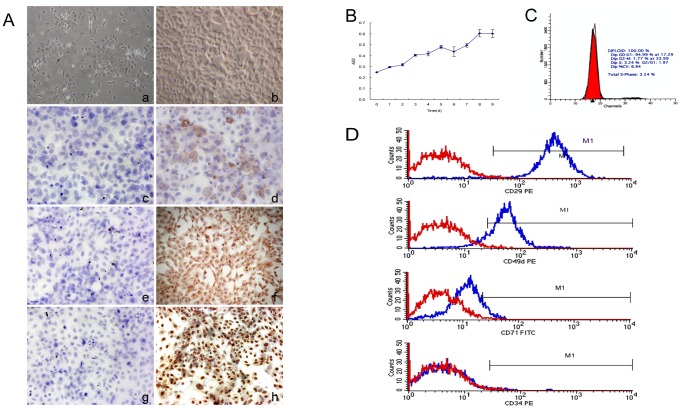
Biological characteristics and verification of rat ESCs. (A) The unique morphology of rat ESCs and cell colonies: (a) ESC colonies formed at d3 under an inverted microscope (100×); rat ESCs have a round or polygonal morphology; (b) ESCs morphology under the phase contrast microscope at d5 (200×), a large flakiness appearance can be seen, characteristic of ESCs; (c) Immunocytochemistry of CK15+ negative control shows no brown granules after staining (100×); (d) Positive ESCs visualized by brown granules: CK15+ expression in the cytoplasm (100×); (e) Immunocytochemistry of CK19+ negative control; (f) Positive ESCs visualized by brown granules: CK19+ expression in the cytoplasm (100×); (g) Immunocytochemistry of p63+ negative control; (h) Positive ESCs visualized by brown granules: p63+ expression in the nucleus (100×); (B) ESCs growth curve; (C) Analysis of cell cycle of rat ESCs showed 95% were in stages G0–G1; (D) ESCs phenotypes identify CD29+, CD45d+, CD71− and CD34− by flow cytometry.

Primary rat epidermal stem cells (ESCs) were isolated from Sprague Dawley rats by using dispase II and trypsin and cultured on type IV collagen. Identification of these ESCs was determined by the unique biological characteristics and marker expression (Figure 1). (Figure 1A) ESC colony formation was observed after three to six days of culture, and ESCs can be identified by their round or polygonal morphological shape (Figure 1A, a). After one to two weeks of culture, ESCs formed a large flakiness appearance (Figure 1A, b).

ESCs were also confirmed by immunocytochemical staining of their unique markers. High levels of Cytokeratin 15 (CK15), Cytokeratin 19 (CK19) and p63 protein were detected in the isolated ESCs (Figure 1A, d,f,h). CK15+ and CK19+ brown particles are expressed in the cytoplasm (Figure1 A, d and f, respectively), and p63+ brown particles are expressed in the nucleus (Figure 1A, h). This is in contrast to the negative control cells which show no brown particles (Figure 1A, c,e,g).

Growth rate of ESCs was examined by Alamar Blue colorimetry (Figure 1B). The growth curve shows that the doubling growth time of ESCs is 48 hours. Furthermore, flow cytometry analysis of the ESCs cell cycle showed that 98% of cells were in the G0–G1 phases (Figure 1C).

ESCs were identified based on their phenotypic expression of β1-Integrin (CD29)+, CD45d+, CD71− and CD34− by flow cytometry (FCM; Figure 1D). The percentage of β1-Integrin, CD45d, CD71 and CD34 in M1 phases is 97.7±5%, 85.03±2%, 8.67±3% and 0.94±5%, respectively. Rat ESCs express CD29 and CD49d, but not CD71 and CD34, indicating that these ESCs were of high proliferation, but low differentiation potential. These results show that ESCs isolated from rat skin could successfully proliferate with ESCs marker.

### Biocompatibility of C-CBM Material and ESCs, and Micro-characteristics of their Co-culture

**Figure 2:**
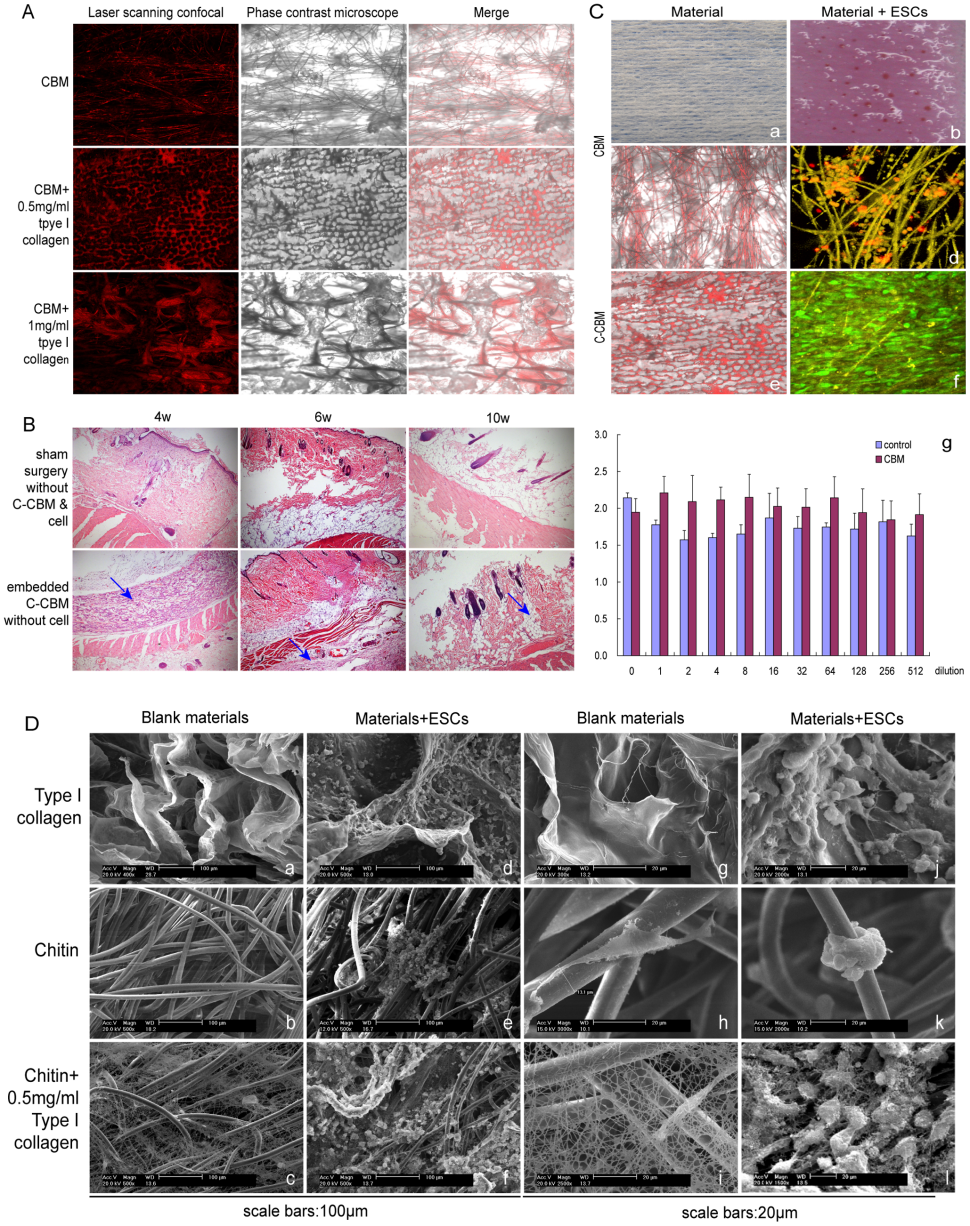
Biocompatibility of C-CBM material and ESCs, and micro-characteristics of their co-culture. (A) Co-culture of ESCs with CBM, CBM+0.5 mg/ml type I collagen, or CBM+1 mg/ml type I collagen. Analysis of CBM modified with different concentrations of type I collagen viewed under a laser scanning confocal microscope and phase contrast microscope (100X). A web-like grid pattern can be seen when CBM is modified with 0.5 mg/ml type I collagen concentration, and is the most suitable environment for ESC growth.(B) The effect of the degradation process of C-CBM in rat full-thickness skin defect models at week 4, 6 and 10. Paraffin and H&E staining methods were utilized. Embedded chitin material (blue arrows) were degraded by week 10 (100X). (C) Biocompatibility of the co-culture of C-CBM or CBM with ESCs. (a) Observation of CBM material with the naked eye; (b) Observation of CBM material with ESCs cell culture with the naked eye; grid-like colonies formed for 2–4 weeks; (c) Pore diameter of non-modified CBM (100X); (d) Pore diameters in CBM with ESCs stained with DiI (100X); ESCs adhered only to junction; (e) Pore diameters in C-CBM material (100X); (f) ESCs-C-CBM stained with GFP (100X); (g) Effect of culture ESCs in media from leaching solution of CBM material. (D) Characterization of cellular behavior on CBM by SEM. Scale bars 100 µm and Scale bars 20 µm. Group 3: Type I collagen, Chitin biomiemtic membrand (CBM) and 0.5 mg/ml type I collagen-chitin biomimetic membrane (C-CBM). (a,g) Type I collagen; (d,j) ESCs grown on the Type I collagen; b,h: Chitin biomiemtic membrand (CBM), (e,k) ESCs grown on the CBM; (c,i) C-CBM, (f,l) ESCs grown on the C-CBM.

Though CBM has been shown to be a successful carrier medium for ESCs, there are still some flaws, such as the uneven spread of ESCs and the weak adherence of ESCs to the covering. The addition of type-I collagen to the membrane carrier (modified CBM) allowed the ESCs to better adhere to the membrane and for greater proliferation. Type I collagen–chitin biomimetic (C-CBM) material was prepared with different concentrations of type I collagen (0.0, 0.5 and 1.0 mg/ml). (Figure 2). Analysis of the different concentrations of type I collagen was done in order to see which preparation was most suitable for growth of ESCs. This was determined by factors such as the abundant spread of ESCs and the production of the smallest pore size in the membrane.

Non-modified CBM (0.0 mg/ml collagen) are electrospun fabric obtained commercially. (Figure 2A, CBM). However, the pore diameter was relatively large (200–500 µm). In contrast, 1 mg/ml modified CBM prevented grid formation because the collagen was too thickly bonded together, and the ESCs also fell off easily after lyophilization (Figure 2A, CBM+1 mg/ml type I collagen). However, if the concentration is too low, the grid formation becomes uneven and the pore diameters are not uniform. In the current study, 0.25–5 mg/ml filtration showed that 0.5 mg/ml type I collagen was the most conducive concentration to grid formation. 0.5 mg/mL modified CBM showed a web-like grid with relatively small pores (2–10 µm in diameter). Thus, the spread of ESCs on this scaffold was distributed fully and evenly (Figure 2A, CBM +0.5 mg/ml type I collagen). Thus, this concentration of CBM showed to be most suitable for ESCs proliferation and differentiation.

The effect of the degradation process of C-CBM in the rat full-thickness skin defect models was also examined to ensure compatibility of chitin with the natural microenvironment of the skin (Figure 2B). In the experimental group, C-CBM was embedded beneath the full-thickness skin post-injury (post-surgery). Sham operations were performed on rats in the control group and contained neither C-CBM nor cells. An inflammatory response ensued two to four weeks after the operation in the group of rats dressed with C-CBM. At six to eight weeks after the initial injury, C-CBM fibers were hydrolyzed, and had a rod-like appearance encircled by connective tissues. At 10 weeks, these fibers were largely degraded and newly repaired tissue took their place. However, granulations appeared in the repaired tissue, though the amount was not significant, especially when compared to the control group. Also, this granulated mass softened over time.

Non-modified CBM was also compared with modified C-CBM (0.5 mg/ml) to evaluate the spread of ESCs on the material. ESCs and either CBM or C-CBM were co-cultured *in-vitro* to further examine the morphology of the ESC culture (Figure 2C). When CBM and ESCs were co-cultured and observed under a laser scanning confocal microscope at two to four weeks, the ESCs were visible on the grid pattern of the dressing (Figure 2C, b). The ESCs were also labeled with DiI. When observed under a fluorescence microscope, ESCs were shown to have adhered to the fiber junctions only. Though there was an affluent amount of colonies, each colony only contained a few cells. Furthermore, the distribution of these cells were uneven over a round or oval area. Also, the adhesion of the ESCs to the material was tenuous and the cells easily fell off. In the middle layer, the original CBM fibers as observed with yellow reflected light showed excellent growth of ESCs (Figure 2C, d). On the other hand, ESCs co-cultured with C-CBM showed more favorable outcomes. ESCs labeled with GFP formed dense colonies on the C-CBM material (Figure 2C, e), and spread widely and evenly. Layered scanning showed that the material surface was covered by cells. Spindle-shaped cells were evenly distributed across the grid, unlike in CBM (Figure 2C, f). Extracting solution of the chitin membrane had no effect on the cells. Comparison between the P3 ESCs group and the control group showed *F* = 0.781, *P*>0.05, indicating no significant difference (Figure 2C g). The secretion of the extracellular matrix was also not significant.

ESCs were observed under a Scanning Electron Microscope (SEM) to see whether or not the ESCs spread sufficiently in their respective carrier mediums (Figure 2D). Pure type I collagen is a good culture substrate for ESCs (Figure 2D, a,d,g and i) and many cell types. But it can dissolve in cell culture media only be maintained for a maximum of three days. This is relatively short compared to type I collagen modified CBM, which showed large amounts of proliferating ESCs and incessancy for at least 4 weeks (Figure 2D, c,f,i and l). Furthermore, C-CBM material showed densely porous wire-mesh structures, evenly distributed, with pore diameters of the mesh aperture ranging 2–10 µm (Figure 2D, i), compared to the unmodified chitin which produced pores ranging in size from 200–500 µm (Figure 2D b). Also, it was observed that the extracellular matrix secreted by cells was woven into the natural mesh grid together with the collagen-modified grid (C-CBM). Cells were evenly spread over the grid and actively growing with vigorous secretion of the extracellular matrix and increased proliferation over controls and clear evidence of mitosis (Figure 2D.f).

These results indicated that the modified chitin membrane was fundamentally a better surface structure for the culturing of ESCs. Fresh rat tail type I collagen mixture was prepared to a final concentration of 0.5 mg/ml type I collagen and a pH 6.9. It will be assumed from this point on that C-CBM refers to a concentration of 0.5 mg/ml of type I collagen.

### Re-epithelialization and Re-growth of Skin Appendages in the Wound Area

*In vivo* experiments were carried out to compare the effects of C-CBM and ESCs-C-CBM in nude mice with full-thickness skin defects. Factors that were evaluated include the presence of early biomarker expression and wound healing capability by immunohistochemical (IHC) assay.

**Figure 3:**
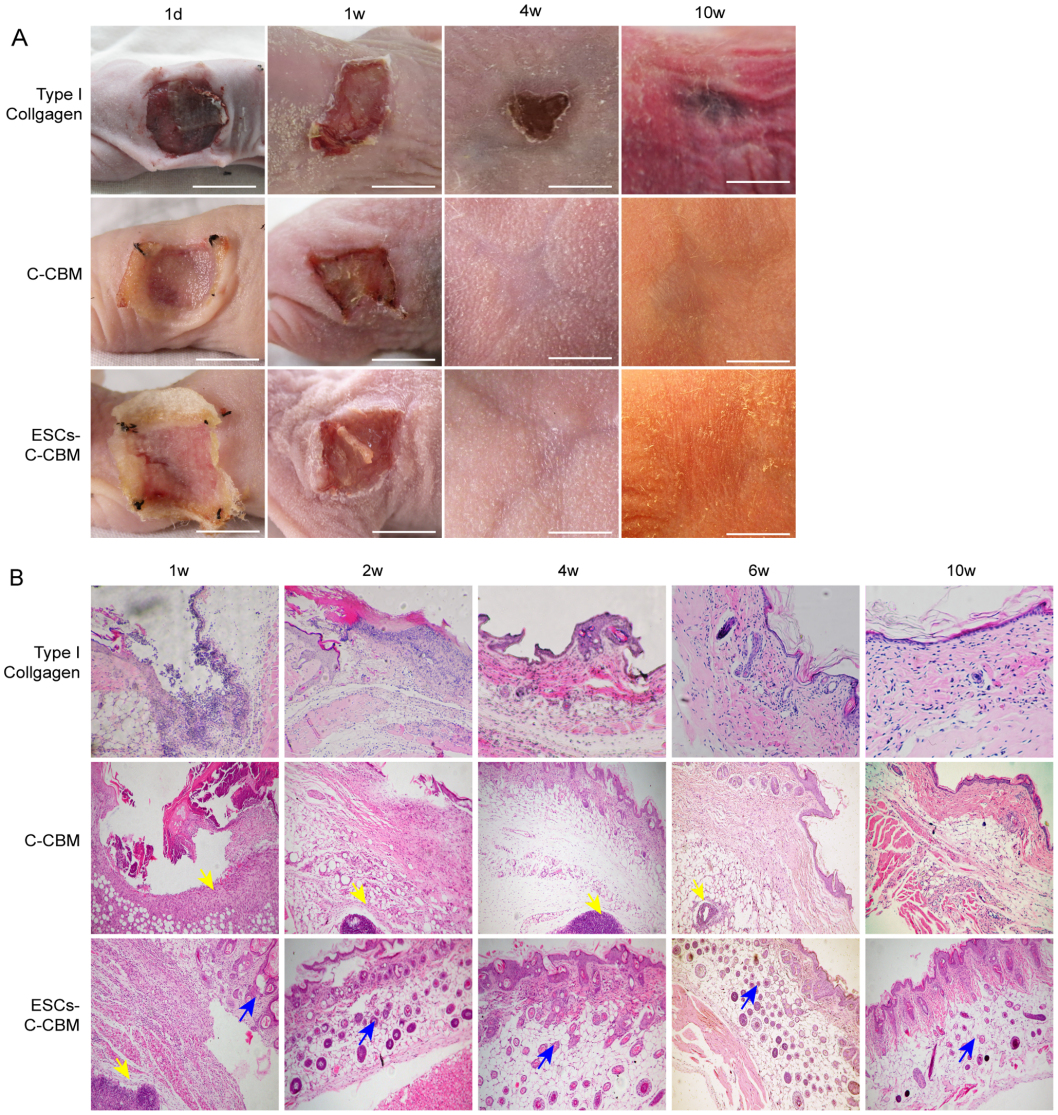
Observation of nude mice full-thickness defect model. (A) Observation of nude mice full-thickness defect model dressed with type I collagen only, C-CBM, or ESCs-C-CBM at 1 d, 1 w, 4 w, and 10 w. Type I collagen group shows that wounds are much slower to heal. Group C-CMB shows the repaired wound skin in the control group was relatively thin and heliotrope with a tendency to bleed; Group ESCs-C-CMB shows the repaired wound in the experimental group was relatively thick and red with re-epithelialization. Scale bar = 1cm. (B) Formation of epidermal nests on the wound surface repaired by epidermal stem cells- collagen-chitin biomimetic (ESCs-C-CBM) membrane compared with C-CBM. Paraffin and stained with hematoxylin and eosin (H&E) stain. Yellow arrows point to chitin and blue arrows point to epidermal nests increased in the ESC-C-CBM group at 2–10 w (100X).

The nude mice were divided into two groups: (1) C-CBM group and (2) ESCs-C-CBM group, and observed for up to ten weeks post-injury. Nude mice wounds dressed with ESCs-C-CBM produced new skin that was relatively thicker, redder and more elastic (Figure 3A). The thickness of the skin in the repaired wound could be observed by the naked eye. The morphology of the repaired skin in this group was similar to that found in unwounded or peripheral tissue, which was soft and red. On the other hand, the control group (C-CBM), The reconstruction skin only formed a thin layer of epidermis, the repaired wound was thin, light purple and had a tendency to bleed easily. When only type I collagen was applied, the wounds took a much longer time to heal and had a dark purplish appearance and wound shrinking.

**Figure 4:**
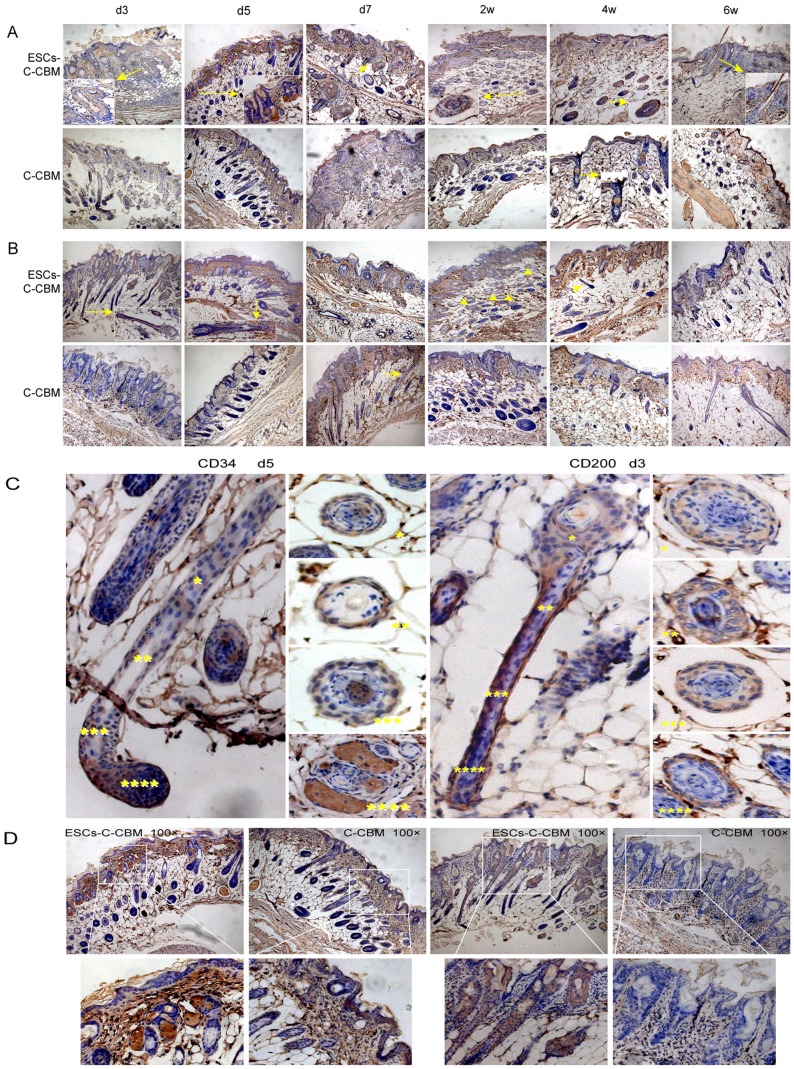
CD34 and CD200 were early-stage markers of hair follicle stem cells by Immunohistochemistry (100X). (A) ESCs-C-CBM shows CD34 expressed in the hair follicle bulge cells at d3 after surgery and maintained from d3 to 4 w, and then decreased after 6 w compare to group C-CBM, which shows CD34 was expressed 4 w after surgery; (B) CD200 was located at the hair follicle group ESCs-C-CMB. It was expressed at d3 after surgery and maintained until 6w compare the control group C-CMB which CD200 was expressed at d 7 and the expression decreased from 4w. (C) Measurement of early-stage *in vivo* CD34/CD200 markers of hair follicle stem cells in the epidermal nests. Hair follicle longitudinal sections and hair follicle transverse sections are both shown. (D) CD34 and CD200 present on ESCs during early stage *in vivo*. CD34 was strongly expressed in the hair bulb at d5. CD200 was highly expressed at the outer hair root sheath at d3.

Furthermore, *in vivo* experiments showed more obvious hair follicle cell proliferation in the full-thickness skin defect nude mice dressed with ESCs-C-CBM compared to those dressed with C-CBM and type I collagen alone (Figure 3B). Haematoxylin Eosin (H&E) staining showed no significant inflammatory reaction in the dermal connective tissue under microscopic examination in the both groups. Also, in ESCs-C-CBM, formation of round or oval areas of net-like epidermis (blue arrows) increased regularly from the second week, was most intense at four to six weeks, and began to reduce after eight to 10 weeks post-injury. Moreover, the net-like epidermis was detected by CD200 and CD34 in wound repair by IHC (Figure 4A–D). The round or oval areas of net-like epidermis were actually different cross-sections of hair follicle cells. Reassembly and analysis of those various sections provides a complete diagram of the hair follicle structure. These events were absent in the control group (C-CBM).

CD34 and CD200 are early-stage markers of hair follicle stem cells. The group in ESCs-C-CBM had more CD34 and CD200 positive cells than the group in C-CBM. Figure 4A shows that CD34 was mainly expressed in the hair follicle bulge cell group in ESCs-C-CBM. This expression occurred mainly at day three after surgery and was maintained from day 5 to week 4, then decreased after six weeks. However, CD34 was expressed up to week 4 in the group C-CBM. These results indicate that ESCs were highly proliferative before week 4 without differentiation and maintained their ESCs characteristics.

Figure 4B shows that CD200 was located at the hair follicle in the ESCs-C-CBM group. It was expressed at day 3 after surgery and maintained until the sixth week, which is relatively a long duration. In the group C-CBM, CD200 was expressed at day 7 and the expression started to decrease after the fourth week, which suggests that the cells already started to differentiate, and the phenotypic expression of CD200 is decreased. CD34 was relatively strongly expressed at the hair bulb at day 5, while CD200 was significantly expressed at the outer hair root sheath at day 3. (Figure 4C, D).

**Figure 5:**
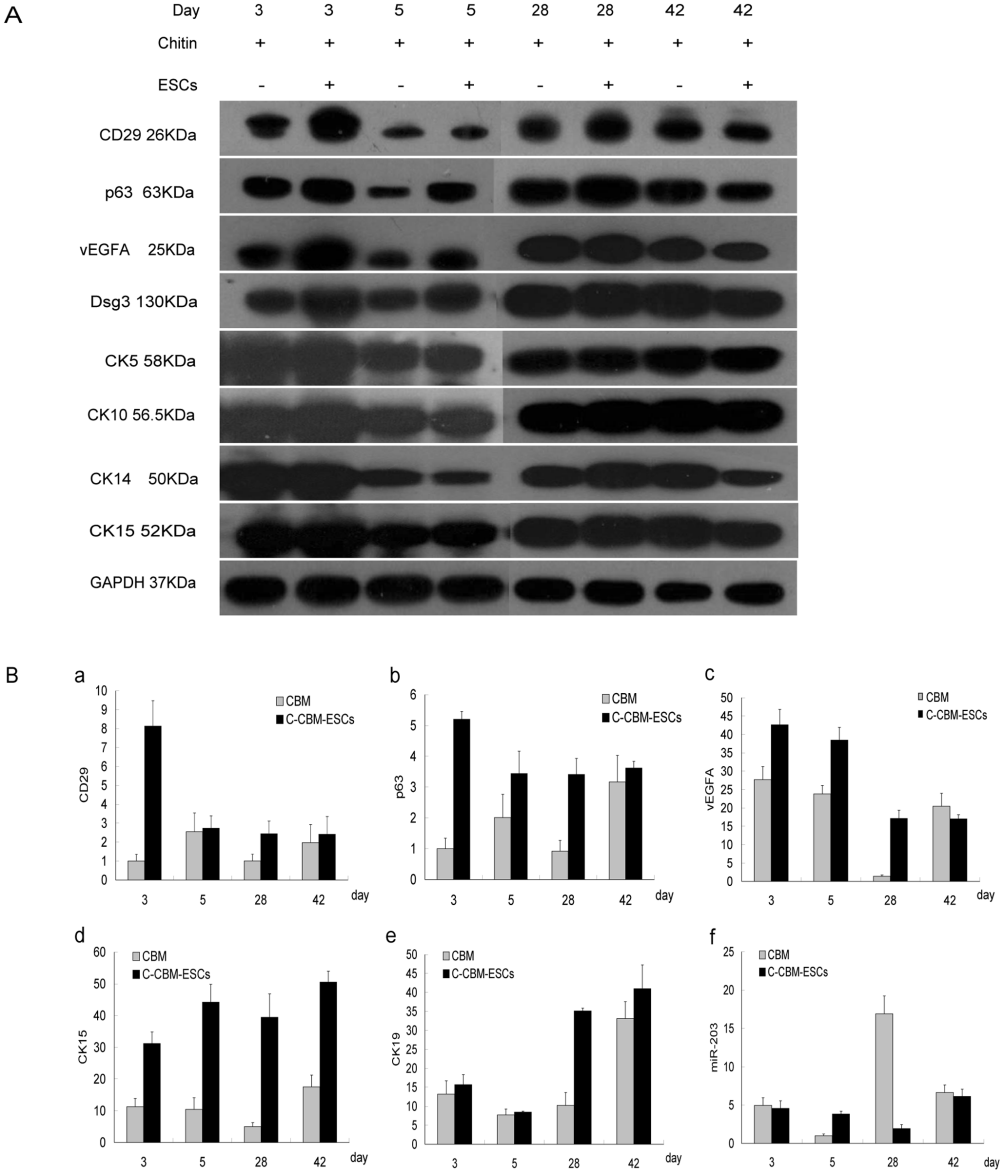
Early-stage markers of protein level and m-RNA level of epidermal stem cells (ESCs). (A) Western blot analysis to detect marked protein. (B) Quantitative real time polymerase chain reaction (RT-PCR) analysis of m-RNA levels: (a) CD-29 increased at d3; (b) p63 was maintained at a relatively high level; (c) VEGF increased at d3–5; (d) CK15 was maintained at a relatively high level; (e) CK-19 was still highly expressed at d28; (f) miR-203 was significantly low at d28 compared to the control group, *P<0.01.

To further examine the early protein expression and mRNA in the wound samples, Western blots and Quantitative RT-PCR were performed. Analysis of these tests showed early protein expression markers were significantly increased; the levels of CD29, p63, VEGFA and Dsg3 at day 3 of ESCs-C-CBM were much higher than those found in the C-CBM group (Figure 5A). Results showed that the ESCs were viable *in vivo* and became active. On the other hand, there were no significant differences between the two groups in the expression levels of transit amplifying cells (TAC) markers in CK5, CK10, CK14 and CK15.

The hair follicle mRNA markers: CD29 (Figure 5B, a), transcription factor p63 (Figure 5B, b) and VEGF-A (Figure 5B, c) and CK15 (Figure 5B, d) were highly expressed in group ESCs-C-CBM at day 3 compared to the group C-CBM without ESCs. Of these, the sustained expression of high levels of CK15 was most noticeable; expression increased three-fold at day 3, four-fold at day 5, eight-fold at day 28, and was continued to increase by a 2.5-fold up to day 42. Moreover, p63 mRNA group increased five-fold over ESCs-C-CBM than group C-CBM, which was maintained until day 28. In addition, CD29 mRNA expression, suggesting early-stage transcription, increased eight-fold at day 3. VEGF-A was highly expressed from day three to 28, and decreased to normal by day 42. CD19 was highly expressed at day 28. Regulatory factor miR-203 did not show a significant difference between the two groups before day 7, but its expression increased in the group C-CBM at day 28 and the two groups significant difference (*P*<0.01) (Figure 5B, f).

## Discussion

The isolation and culture of ESCs *in vitro* and creation of a three-dimensional (3D) tissue skin structure *in vitro* for their clinical utilization has been a challenge for clinical application in its advent to improve the process of wound healing [25]. Kojma *et al* suggested that chitin was able to stimulate endogenous collagen production [26]. Currently, chitin membrane has been used as wound coverings in clinical practices. However, the addition of ESCs to this membrane, as well as modifying the membrane with type I collagen, has not been done before. The objective of this study was to see whether chitin with ESCs culture and exogenous collagen-modification would be a more effective wound covering than just chitin itself, and to observe whether such wound covering would be in accordance with the natural microenvironment required for proper wound healing.

First, we described a procedure for isolation and the culture of ESCs (with its markers) from a rat’s skin’s backside (Figure 1). Then, we compared two different cell scaffolds: (1) CBM and (2) C-CBM. CBM is chitin membrane without type I collagen added. It is purchased from commerce and has been pre-electrospun. The other type, C-CBM, is coated chitin that has been modified with type I collagen. In a previous experiment, Lee coated type I collagen to the surface of chitin scaffold and used sodium chloride, and showed that it produced large pores that were 260–330 µm in diameter [27]. Results showed that 0.5 mg/ml collagen concentrations produced a patterned web-like grid (Figure 2A) with pores as small as 2–10 µm in diameter. Thus, cells were able to spread fully and evenly throughout the matrix. This is in contrast to CBM, which contained pores that were 200–500 µm in diameter (Figure 2C c). The implanted fibroblasts showed relatively good affinity and proliferative ability after 14 days. If the collagen concentration is too high and the bonded powder too thick, cells easily fall off. If the collagen concentration is too low to form a grid or the mesh diameter is too large, the cells cannot adhere well.

Second, we compared type I collagen coated on the surface of chitin scaffold (C-CBM) with and without ESCs *in vivo* on nude mice. Patterned surface modification affects the relationship of cells with each other, the extracellular matrix, and soluble factors to change the differentiation and modification of stem cells. Improving the biological induction material requires increasing the contact between the cells and the surface or the interface of the material, forming a “bio-mimetic surface” with a microenvironment that optimizes the distribution and reconstruction of such active materials as the extracellular matrix and cytokines for growth.

Furthermore, to explore the use of C-CBM with implanted ESCs for wound repair we examined the effects of cell differentiation. Previous experiments showed that the chitin membrane was a suitable carrier medium [28]. The C-CBM produced *in vitro* use of ESCs for a wound covering *in vivo* and helps ESCs survive on the wound surface. C-CBM is degraded automatically 2–3 weeks after application and becomes detached. Figure 3B shows numerous round and oval epidermal nests formed under the skin of *in situ* repair in the animal model of full-thickness skin defects. These nests may be related to replication and migration, during which ESCs differentiate into epidermal cells. A single application of ESCs without C-CBM support will not result in the cells entering the wound surface because the leaked tissue fluid quickly removes them. As an important medium, collagen can be combined with integrins in ESCs to produce dense connections between cells. C-CBM was applied in the current study to significantly increase the rate of proliferation of ESCs, and was interwoven into a network with the extracellular matrix secreted by the cells. With regard to early-stage protein expression, we found increased m-RNA transcription in CD29, p63, VEGF-A, sustained expression of high levels of CK15 and low levels of the regulatory factor miR-203, suggesting that the chitin membrane promotes early-stage protein synthesis at the wound surface and enhances the healing speed together with ESCs which is play an important role in this process, perhaps because the signaling molecules secreted by these cells may induce ESCs differentiation, resulting in self-repair and regeneration of tissues after activation.

Clinically, the formation of normal re-epithelialization and follicular orifices is very important for skin wound repair. The follicular orifice is used for sweat gland secretion, and also speeds up wound healing and reduces scar formation that raise the quality of wound healing. Additionally, hair follicular stem cells (FSCs) play a major role in this process. It is well-known that cytokeratin 15 (CK15), CK19, CD29, CD200 and CD34 are highly expressed surface markers of hair FSCs, which correlated with our data. These results indicated that the surface markers of FSCs were present. The others include α6-integrin (CD29), CD71, CK19, CK15, CD34 [29], [30], [31]. Because CD29 is highly expressed on the surface of and transiently proliferating cells, but not in post-mitotic and terminal cells, it can be used to differentiate ESCs and transiently proliferating cells. CD71 is a transferrin receptor on the surface of ESCs. Some epidermal cells with low levels of CD71 have the characteristics of ESCs. Relatively strong expression of CK15 was previously observed in mouse ESCs [32]. CK15 and Kl9 are keratins expressed at the early stage of cell differentiation. During FSC differentiation, CK15 expression is reduced earlier than that of CK19, suggesting that decreased CK15 expression may be the earliest sign of cellular differentiation of transiently proliferating cells. Cells with negative CK15 and positive CK19 may be “early-stage” transiently proliferating cells. CK15 may therefore be a more meaningful marker of FSC differentiation than CK19. In the current study, cytokeratin 15 is a specific marker of stem cells of the hair-follicle bulge expression of CK15 continued at day 28, implying that the characteristics and function of ESCs may be maintained for 28 days in this microenvironment and the transiently proliferating cells appear relatively late. CKl9 is scattered at the basal layer of the epidermis and deep in the reticular spine in accordance with the location of label-retaining cells (LRCs). CK19 positive cells within the bulge are LRCs and slow cycling stem cells [33]. Advances now indicate there are a number of stem cell repositories within the epidermis, two of which, the interfollicular epidermis and the bulge region of the hair follicle, may supply each other when damaged [34].

CD34 is a specific marker of hematopoietic stem cells, as well as a phenotype marker of vascular endothelial cells. Trempus demonstrated that CD34 is a special marker of skin bulge cells, showing it to be highly expressed in the bulge [35]. CD34 and K15 positive cells are located at the same place in the rat hair follicle, but K15 expression is decreased or absent in the bulge of human hair follicles [36]. Therefore, CD34 is also a marker of rat ESCs and vascular endothelial cells.

CD200 is a recently-found marker of ESCs. Most residual cells with slow cycling markers are located at the hair follicle bulge, CD200 positive cells showed greater ability to form clones [37] and CD200 is expressed in cells on the outer root sheath of the hair follicle [38]. This niche microenvironment found in the hair follicle’s bulge possesses intrinsic “stemness” features without restricting the establishment of epithelial polarity or changes in gene expression [15]. Thus, bulge cells can develop into epidermal stem cells.

During rodent experiments, the speed of wound healing of the hair follicle was quicker at the anagen period than at the telogen phage. In autologous skin graft surgery, the scalp can usually be used as the donor site for repeated skin harvesting without scar formation. This practice suggests that the hair follicle is closely related to wound healing. Taylor et al. reported that FSC can not only differentiate into various types of cells within the hair follicle, but also differentiate into skin epidermal cells during the periodic cycle of the hair follicle [17]. Tumbar et al. studied histone-GFP mice to show that GFP positive cells from the bulge differentiated into the outer root sheath of the hair follicle, hair and inner root sheath cells at the anagen phase [39]. Moreover, labeled slow cells moved out from the bulge, migrated and differentiated into the basal membrane and epidermal cells when the skin was injured. After labeling and implanting isolated dermal cells of the rat hair follicle in wounds in the rat ear and back, researchers observed that the labeled cells took part in the repair of dermal tissue, acting just like fibroblasts taking part in wound healing [40]. Amoh et al. also found in rat experiments that some parts of new capillaries originated from labeled cells in the anagen phase of the hair follicle during skin wound repair [41], [42]. These results suggest that FSC may take part in dermal vascularization during wound healing. FSCs can differentiate into sebaceous glands or epithelial cells in the sweat gland [43]. As a transcription factor, p63 is related to the genotypic change of stem cells or transiently proliferating cells. A homolog of p53, it is localized in the nucleus and is involved in epidermal development. It plays a decisive role in maintaining the biological properties and proliferation and differentiation of ESCs [44].

MiR-203 is a regulation gene of ESCs differentiation. If expressed too early in epidermal cells in the basal layer, miR-203 may result in premature differentiation and defects of proliferative potential. MiR-203 expression is obvious during epidermis differentiation and development. Moreover, it evolves into conservative miRNA. MiR-203 targets Np63 mRNA, and acts as a switch in the proliferation and differentiation of keratinocytes in the adult epidermis. Therefore, miR-203 is a key molecule controlling the differentiation of keratinocytes from the basal state to the basal layer. MiR-203 regulates p63 by suppressing translation, and strongly inhibits p63 expression. After the defects of miR-203, p63 translation in the basal epidermis will increase. Over-expressed miR-203 reduces Np63 mRNA [45]. It has been reported that p63 expression is strongly inhibited during low-calcium culture of primary mouse keratinocytes transfected with wild-type miR-203, which indicates that miR-203 regulates p63 by translational suppression [46]. Although VEGF-A is an important regulatory factor of carcinogenesis, it is also expressed in certain normal tissues. Angiogenesis during embryonic development depends on normal VEGF-A expression. Neovascularization is needed during wound repair and wound healing may be affected by abnormal VEGF expression [47]. In the present study, VEGF-A disappeared after d28, suggesting that increased VEGF-A production is important during early phase wound healing, then turns off subsequently.

The present study showed that ESCs produced *in vitro* with the related scaffold can form a temporary biomimetic covering. ESCs can differentiate into epidermal cells to enhance wound healing, inducing *in situ* regeneration of nude mice full-thickness skin defects. This shows that biological material containing seed cells can promote wound healing. However, the final turnover of the epidermal nests should be further identified by labeling. Simulation experiments of the structure and function of the ESCs microenvironment showed production of biological induction materials imitating the natural extracellular matrix with signaling molecules effectively regulating the differentiation of ESCs to stimulate and induce self-repair and regeneration. The result was the morphological and structural regeneration and functional reconstruction of damaged tissue.

## Conclusion

Our findings that 0.5 mg/ml rat tail type I collagen can be used for surface modification of the chitin membrane, creating a relatively good biocompatible material Cells secrete various bioactive substances, which closely cooperate and coordinate with each other and its surrounding microenvironment. ESCs grow well on C-CBM and are important in wound repair. The results of both *in vivo* and *in vitro* experiments showed ESCs were highly spread over the C-CBM surface and the proliferation rate increased significantly. ESCs-C-CBM plays a role in induction of hair follicular stem cells (FSCs) and are the main ESCs growing on the modified chitin membrane. Reconstruction of the epidermal and the dermal layer could be observed in full-thickness skin defects in an *in vivo* experiment, with more hair follicle stem cells, and the skin on the repaired wound was relatively thick and red with clear signs of re-epithelialization.

Hair FSCs can differentiate into epidermal cells and skin appendages to achieve initial repair of the epidermis and dermis without having to engineer both skin tissues separately. It can be applied to both superficial and full-thickness wound repair in nude mice. ESCs-C-CBM produced by tissue engineering using this matrix grid can reconstruct tissues with structures and metabolic activities similar to the natural skin in a relatively short period, and is an important candidate for clinical wound repair for such injuries as burns, skin wounds and diabetic foot ulcers.
